# Supplementary material for: Low-carbohydrate diets differing in carbohydrate restriction improve cardiometabolic and anthropometric markers in healthy adults: A randomised clinical trial
Source: PeerJ. 2019 Feb 5;7:e6273. doi: 10.7717/peerj.6273 (PMC6368026; doi:10.7717/peerj.6273)
Supplement: Supplemental Information 3 [file peerj-07-6273-s003.docx]

**Public Title:**

How low do you need to go? Comparing symptoms of diet induction and mood with outcomes from diets containing differing levels of carbohydrate restriction.

**Working Title /Scientific Title:**

Does a moderate carbohydrate restriction result in fewer symptoms of dietary induction and greater maintenance of mood, with comparable cardiometabolic results, when compared to more extreme carbohydrate restriction in healthy volunteers?

**Short Working Title:**

Comparing cardiometabolic outcomes to symptoms of diet induction and mood in low carbohydrate diets differing in the level of carbohydrate restriction.

**Summary**

A 13-week, 3-arm, randomised trial comparing diets consisting of 5, 15, or 25% calories from carbohydrate, 20% calories from protein and the remainder from lipids. Primary outcomes are change from baseline of symptoms and mood, as measured by a symptoms questionnaire (Symptoms-Q), and Profile of Mood States (POMS-SF), taken on awakening, fasted. Secondary outcomes include change in cardiometabolic (CM) status as measured by change from baseline triglyceride (TAG) to HDL ratio; and TAG, total/LDL/non-HDL/HDL cholesterol, anthropometry (BF%), HbA1c, serum urate, and c-reactive protein. A one week lead-in will be used to determine baseline calories, and mood and symptoms scores, for comparison to completion measures. Calories from the lead-in week will be maintained throughout the study. Symptoms and mood questionnaire will be taken daily, along with BOHB for the first 20 days. CM measures will be repeated upon completion. Symptoms and mood scores will be aggregated and summarised into mean changes from baseline and analysed by treatment group using ANOVA and compared with multiple linear regression. For secondary outcomes, changes in biomarker concentrations and anthropometry will be analysed using ANOVA and multiple linear regression. Tests will be two-tailed and the type-1 error rate of 5% will be used to judge statistical significance.

**Background**

Reduced carbohydrate diets offer benefits for the prevention and adjunct treatment of health conditions ranging from neurological disorders, cancer, obesity, diabetes and other conditions on the spectrum of metabolic syndrome.^1-11^ They are becoming increasingly popular for mainstream and athletic use for a range of outcomes including weight-loss and maintenance,^12^ and have been demonstrated to improved satiety and reduce hunger.^13-15^ But adaptation from a standard diet to one low in carbohydrates can cause various unpleasant symptoms,^16^ referred to in common parlance as ‘keto-flu’. These symptoms of carbohydrate restriction are assumed to provide a barrier to the use of, and compliance with low carbohydrate diets. However, these early-induction symptoms have not been well described in the scientific literature.

So, at this time, it is not clear whether symptoms are related solely to keto-induction (i.e. in very low carbohydrate ketogenic diets) or are more generally, symptoms of carbohydrate withdrawal. Outcomes from ketogenic and non-ketogenic lower carbohydrate diets are likely to be similar^17^ and the degree to which people experience symptoms of carbohydrate withdrawal or keto-induction is also likely to influence compliance. For many, it may not be necessary to restrict carbohydrate to the degree necessary to achieve ketosis, especially if the symptoms resulting from extreme carbohydrate restriction are disproportionate to the beneficial outcomes from the diet. This has not been investigated and this study will seek to address this important clinical topic

**Study Hypothesis:**

A moderate restriction of carbohydrate results in fewer symptoms of dietary induction and greater tolerance and adherence to diet, with comparable results for cardiometabolic outcomes when compared to more extreme carbohydrate restriction.

**Primary outcome:** Symptoms and mood

**Primary outcome measures:** Daily Symptoms-Q, sum of symptoms score; and POMS-SF, total mood disturbance score (TMDS), taken on awakening, before breakfast. Change from baseline.

**Covariates/Potential confounders:** Gender, age, BMI, BF%, baseline CHO/sugar intake, medications, medical conditions, smoking status, alcohol intake.

[i.e. as related to hypothesis; symptoms of keto-induction / carb-withdrawal are going to be lower in the moderate group than in the more severe]

**Secondary Outcomes:** Change in cardiometabolic markers

**Secondary outcome measures:** Primary measure for secondary outcome: Change from baseline (TAG/HDL ratio). Also: triglycerides, total/LDL/non-HDL/HDL chol., anthropometry (BF%), HbA1c, serum urate, CRP

**Methods**

A randomised, three-armed, single blinded, trial.

Data to be collected at AUT Millennium

**Recruitment**

144 participants will be recruited from the networks of the four researchers, including from Auckland University of Technology at Millennium Institute of Sport and Health (AUT-MISH) (Albany, New Zealand) to participate in the trial. As the focus of this research is not ‘sensitive’, a ‘snowball recruitment’ approach is appropriate, encouraging applicants to share the advertisement for participants amongst their respective networks (for example via social media).^18^

The power calculation for participant numbers is based on the findings of the pilot study: *The effect of medium chain triglycerides on time to nutritional ketosis and symptoms of keto-induction in healthy adults* (manuscript submitted for publication). The primary outcome chosen for this power calculation is change in POMS-TMDS from baseline. This measure was chosen as a validated questionnaire that is fit for our purpose of evaluating the effect of dietary induction on participants. In the pilot study the mean POMS-TMDS was -11.4 in the control group (SD: 17.3) and -5.1 (SD: 7.2) in the experimental. We expect to see lowest mood disturbances in the NKLCD arm of the study compared to the VLCKD arm. Assuming the ketogenic arm has a change from baseline of 11.4 (SD 17.4) and the minimum change in the NK arm is +9.2 units higher with a restricted SD, as seen in the pilot (SD 7), then 36 participants per arm is required to detect this difference with a false positive (α) rate of 5% and false negative of (β) 20%. We will allow for 48 per group to allow for dropouts based on pilot.

Criteria for inclusion: Non-obese, non-diabetic, between the ages of 25 and 49.

Exclusion: BMI <19 or >30, diagnosed metabolic condition such as diabetes, or cardiovascular disease, chronic kidney or liver disease. Pregnancy or breastfeeding. Habitual intake of >40% calories from carbohydrate.

**Lead-in week**

A one-week lead-in phase will be carried out, in which participants will record dietary intake using a phone and web-based application, ‘Fat Secret’ Professional’, and complete a daily questionnaire and record fasting blood BOHB and the online questionnaire described below, on awakening. Participants will be instructed to not substantively change activity levels for the duration of the entire study period, and thus, differences in activity will be accounted for by the baseline information collected. The lead-in phase will provide baseline characteristics of the habitual eating of the participants and of their mood-state and any symptoms they are currently experiencing. It will also familiarise the participants with the blood-prick measures, online questionnaires and the use of the food tracking app.

**Randomisation**

Participants will be allocated into one of three groups by a randomised sequence prepared by the study statistician and given a sealed envelope with their diet allocation.

**Intervention groups**

After the lead-in, participants will be assessed at baseline for:

- Blood lipid and cardiometabolic profile including TAG, total cholesterol, high-density lipoprotein (HDL-C), low-density lipoprotein (LDL-C), c-reactive protein (CRP), glycated haemoglobin (HbA1c).
- Anthropometric assessments; weight, waist girth measurement, hip girth measurement, waist to hip ratio.

Participants will be instructed on the dietary interventions by a qualified and registered clinical nutritionist and dietitian, with meal plans provided. Blood measures and anthropometric assessment will also be carried out at the conclusion of the study for comparison to baseline measures.

Average daily calorie intake per person will be calculated from data collected during the lead-in phase and this intake will be maintained over the study duration. Participants will continue to track their diets using ‘Fat Secret Professional’ for 20 days, attempting to meet targets for carbohydrate, protein and lipids based on their prescribed diets. Diets will consist of the following targets, individualised to each participant in grams per day, based on their baseline, habitual calorie consumption.

|  | Protein | Carb | Fat |
| --- | --- | --- | --- |
| Very Low Carbohydrate Ketogenic Diet | ~20% | 5% | ~75% |
| Modified Ketogenic Diet | ~20% | 15% | ~65% |
| Non-ketogenic Low Carbohydrate Diet | ~20% | 25% | ~55% |

Participants will be provided with a nutrition plan and calorie and macronutrient prescription but they will also have freedom to choose meal timing and frequency, so long as they are adhering to the calorie and macronutrient guidelines in their plan.

Blood BOHB measures will continue to be taken daily for the first twenty days of the study, in the morning upon waking (fasted). Participants will also complete a daily questionnaire in the morning upon rising, consisting of a ketogenic diet questionnaire (Symptom-Q) previously developed by the author and Profiles of Mood States, short-form questionnaire (POMS-SF), for the entire study duration, along with daily weight measure. They will also be instructed to continue to continue where able, to track daily calories.

From day 20 onwards, participants will be instructed to continue their carbohydrate allocation, as a percentage of ad libitum calories, as best able.

Explanation of daily measures:

**Beta-hydroxybutyrate (BOHB).** Participants will be provided with a ‘FreeStyle Neo’ blood-prick ketometer/glucometer (Abbott Industries). Participants will be required to use the device (by way of a personal blood prick) to measure and record BOHB daily upon waking. Taken for the first twenty days.

**Online survey.** Indicators of general and psychological health and subjective symptoms of carbohydrate withdrawal will be evaluated by a daily electronic (online) survey, using Typeform software which will include: 1) a modified 5-point mood scale and 2) a carbohydrate restriction symptoms questionnaire using a Likert Scale (developed by the primary researcher). The *symptoms checklist* will be used to monitor the commonly observed symptoms resulting from carbohydrate withdrawal, namely constipation, headache, halitosis, muscle cramps, diarrhoea, general weakness and rash.^20^

**Data analysis**

Subjects will be compared with regard to baseline variables by treatment allocation arm to check for balance. Exploratory data analysis will consider the variation in different facets of the symptom scores with time from commencing the diet, by treatment arm, and by sociodemographic and clinical characteristics. Correlation between individual items in the score and baseline characteristics will be undertaken using principal components and biplots. Similar analyses will be carried out for associations between. Daily symptoms scores will be aggregated and summarised into mean changes from baseline. These changes will then be analysed by treatment group using ANOVA. These analyses will be compared with multiple linear regression, with baseline characteristics included in the regression model. Similarly, for secondary outcomes, changes in biomarker concentrations and anthropometry will be analysed using ANOVA and multiple linear regression. Linear mixed effects models, with within individual All tests will be two tailed and the type-1 (false positive) error rate of 5% will be used to judge statistical significance.

**References**

1. Lefevre F, Aronson N. Ketogenic Diet for the Treatment of Refractory Epilepsy in Children: A Systematic Review of Efficacy. Pediatrics. 2000;105(4):e46.

2. Keene DL. A Systematic Review of the Use of the Ketogenic Diet in Childhood Epilepsy. Pediatric Neurology. 2006;35(1):1-5.

3. Levy RG, Cooper PN, Giri P, Pulman J. Ketogenic diet and other dietary treatments for epilepsy. The Cochrane Library. 2012.

4. Henderson CB, Filloux FM, Alder SC, Lyon JL, Caplin DA. Efficacy of the Ketogenic Diet as a Treatment Option for Epilepsy: Meta-analysis. Journal of Child Neurology. 2006;21(3):193-8.

5. Neal EG, Chaffe H, Schwartz RH, Lawson MS, Edwards N, Fitzsimmons G, et al. The ketogenic diet for the treatment of childhood epilepsy: a randomised controlled trial. The Lancet Neurology. 2008;7(6):500-6.

6. Paoli A, Rubini A, Volek J, Grimaldi K. Beyond weight loss: a review of the therapeutic uses of very-low-carbohydrate (ketogenic) diets. European journal of clinical nutrition. 2013;67(8):789-96.

7. Sumithran P, Proietto J. Ketogenic diets for weight loss: A review of their principles, safety and efficacy. Obesity Research & Clinical Practice. 2008;2(1):1-13.

8. Maalouf M, Rho JM, Mattson MP. The neuroprotective properties of calorie restriction, the ketogenic diet, and ketone bodies. Brain research reviews. 2009;59(2):293-315.

9. Castro K, Faccioli LS, Baronio D, Gottfried C, Perry IS, dos Santos Riesgo R. Effect of a ketogenic diet on autism spectrum disorder: A systematic review. Research in Autism Spectrum Disorders. 2015;20:31-8.

10. Varshneya K, Carico C, Ortega A, Patil CG. The Efficacy of Ketogenic Diet and Associated Hypoglycemia as an Adjuvant Therapy for High-Grade Gliomas: A Review of the Literature. Cureus. 2015;7(2):e251.

11. Kulak D, Polotsky AJ. Should the ketogenic diet be considered for enhancing fertility? Maturitas. 2013;74(1):10-3.

12. Bueno NB, de Melo ISV, de Oliveira SL, da Rocha Ataide T. Very-low-carbohydrate ketogenic diet v. low-fat diet for long-term weight loss: a meta-analysis of randomised controlled trials. British Journal of Nutrition. 2013;110(07):1178-87.

13. Paoli A, Bosco G, Camporesi E, Mangar D. Ketosis, ketogenic diet and food intake control: a complex relationship. Frontiers in Psychology. 2015;6.

14. McClernon FJ, Yancy WS, Jr., Eberstein JA, Atkins RC, Westman EC. The effects of a low-carbohydrate ketogenic diet and a low-fat diet on mood, hunger, and other self-reported symptoms. Obesity (Silver Spring, Md). 2007;15(1):182-7.

15. Johnstone AM, Horgan GW, Murison SD, Bremner DM, Lobley GE. Effects of a high-protein ketogenic diet on hunger, appetite, and weight loss in obese men feeding ad libitum. The American Journal of Clinical Nutrition. 2008;87(1):44-55.

16. Hartman AL, Vining EP. Clinical aspects of the ketogenic diet. Epilepsia. 2007;48(1):31-42.

17. Johnston CS, Tjonn SL, Swan PD, White A, Hutchins H, Sears B. Ketogenic low-carbohydrate diets have no metabolic advantage over nonketogenic low-carbohydrate diets. The American Journal of Clinical Nutrition. 2006;83(5):1055-61.

18. National Science Foundation. How should research involving "snowball samples" be handled from a human subjects perspective? Arlington, Virginia, USA: National Science Foundation,; N.D [

19. Shacham S. A Shortened Version of the Profile of Mood States. Journal of Personality Assessment. 1983;47(3):305-6.

20. Yancy WS, Jr., Olsen MK, Guyton JR, Bakst RP, Westman EC. A Low-Carbohydrate, Ketogenic Diet versus a Low-Fat Diet To Treat Obesity and Hyperlipidemia: A Randomized, Controlled Trial. Annals of Internal Medicine. 2004;140(10):769-77.
